# Supplementary figures and images for: The structure of photosystem I from a high-light-tolerant cyanobacteria
Source: eLife. 2021 Aug 26;10:e67518. doi: 10.7554/eLife.67518 (PMC8428864; doi:10.7554/eLife.67518)

*C. aponinum*  
WT Synechocystis

WT Synechocystis  
Red\_c  
Red\_d

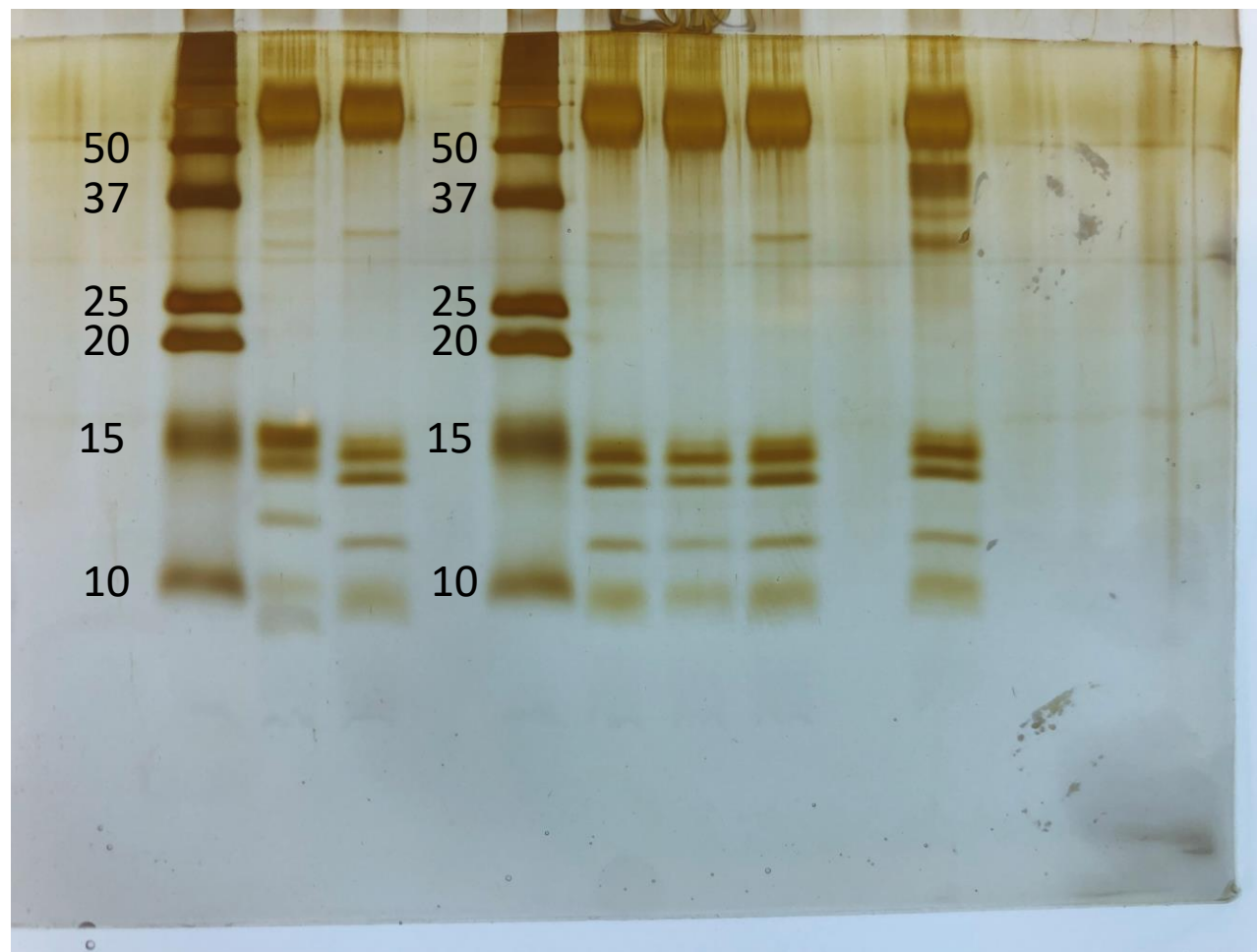

Supplement: Figure 2—source data 1. [file elife-67518-fig2-data1.zip › Figures_2_labeled_Gel_image.pdf]

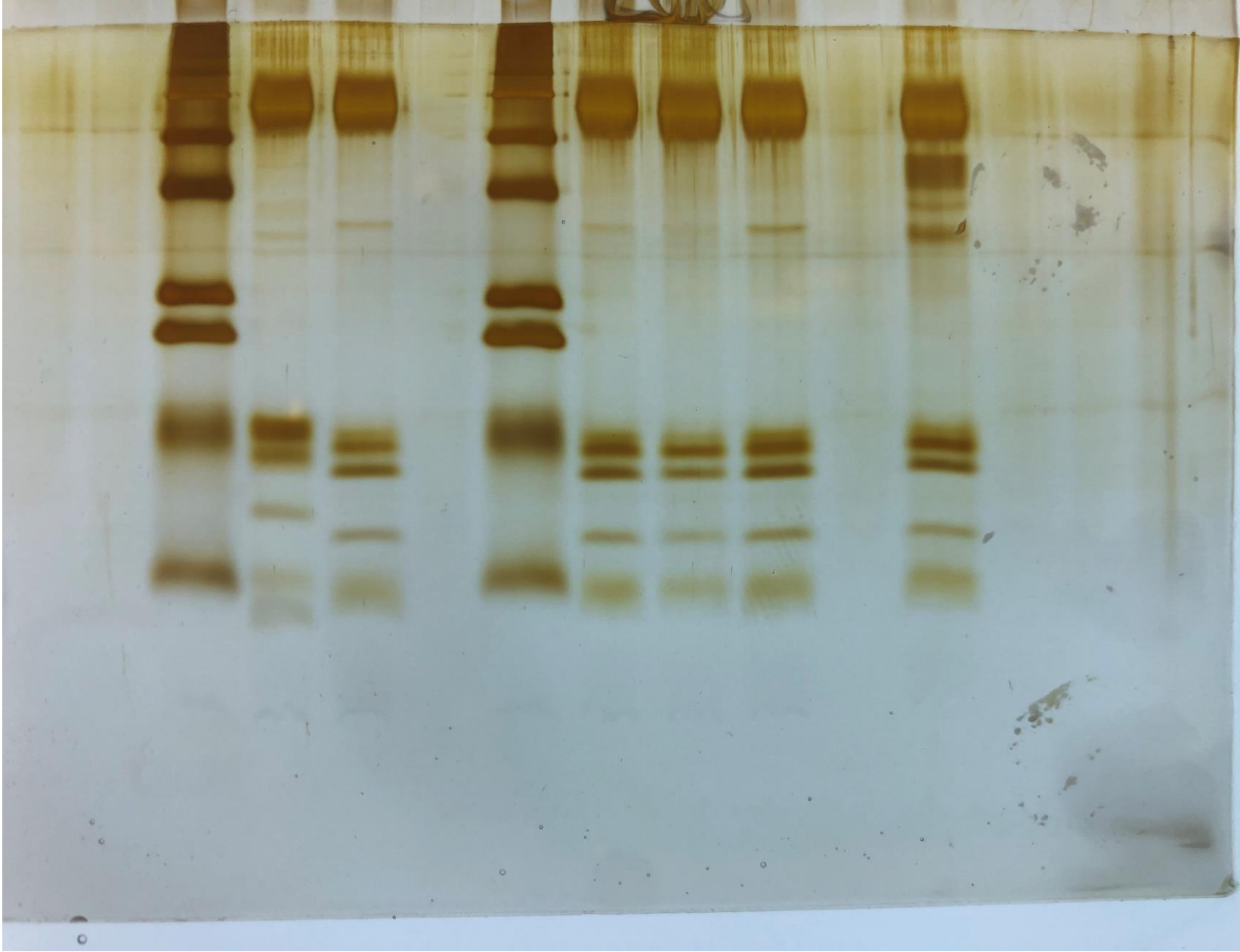

Supplement: Figure 2—source data 1. [file elife-67518-fig2-data1.zip › Figures_2_Raw_Gel_image.pdf]
