# Supplementary material for: The structure of photosystem I from a high-light-tolerant cyanobacteria
Source: eLife. 2021 Aug 26;10:e67518. doi: 10.7554/eLife.67518 (PMC8428864; doi:10.7554/eLife.67518)
Supplement: Supplementary file 1. — Scores were calculated using the MapQ plugin in UCSFChimera. (1b). Primer list used for mutant construction. [file elife-67518-supp1.docx]

**Supplementary file 1a.** Individual chains and ligand resolvability according to Q-scores. Scores were calculated using the MapQ plugin in UCSFChimera.

| **Chain** | **Q Score** | **Expected @ 3.00** | **Estimated Resolution (Å)** |
| --- | --- | --- | --- |
| A | 0.69 | 0.5867 | 2.39 |
| B | 0.69 | 0.5867 | 2.41 |
| C | 0.68 | 0.5867 | 2.47 |
| D | 0.64 | 0.5867 | 2.67 |
| E | 0.65 | 0.5867 | 2.67 |
| F | 0.63 | 0.5867 | 2.77 |
| I | 0.68 | 0.5867 | 2.5 |
| J | 0.66 | 0.5867 | 2.56 |
| K | 0.58 | 0.5867 | 3.05 |
| L | 0.66 | 0.5867 | 2.61 |
| M | 0.67 | 0.5867 | 2.52 |
|  |  |  |  |
| **Average:** | 0.66 | 0.5867 | 2.62 |

**Supplementary file 1b.** Primer list used for mutant construction.

| Primer Name | Sequence |
| --- | --- |
| **P60_red_c_Forward_insert** | GCCGCCCATAATCCCCCCCAGGGCACCCCCTTCGGCGGCGCCTTGACCGGCGCAGGCCATACCAACCTGTAC |
| **P60_red_c_Reverse_insert** | GGCCACAGCATCAAAGAAATTCTTGCCGCCCATAATCCCCCCCA |
| **P60_red_c_Forward** | aacgcagaccgttccgtggcaaagca |
| **P60_red_c_Reverse** | ccgacaacgcagaccgttccgtggc |
| **PsaL_F** | GGCGCAGTTACCCATTTCCGCTG |
| **PsaL_R** | GAGTAACAAATCAAAATGTCCAATC |
| **Cm_F** | GACATTTTGATTTGTTACTCCcgaataaatacctgtgacggaag |
| **Cm_R** | AAAAATACCCCACCCGCTGGCccaggcgtttaagggcaccaataac |
| **Down_F** | GCCAGCGGGTGGGGTATTTTTTTTGTG |
| **Down_R** | GCCATATTATCGAAAAATTCCAGGG |
| **Backbone_F** | GGAATTTTTCGATAATATGGCcacaattccacacattatacgag |
| **Backbone_R** | CGGAAATGGGTAACTGCGCCatgtcatgataataatggtttc |
| **Ca_D2L_F** | GGGCCCGCTGCGtctgTCTGAGTAC |
| **Ca_D2L_R** | GTACTGGTACTCAGAcagaCGCAGC |
